# Ocular manifestations of recent viral pandemics: A literature review

**DOI:** 10.3389/fmed.2022.1011335

**Published:** 2022-09-23

**Authors:** Mohammad J. J. Taha, Mohammad T. Abuawwad, Warda A. Alrubasy, Shams Khalid Sameer, Taleb Alsafi, Yaqeen Al-Bustanji, Luai Abu-Ismail, Abdulqadir J. Nashwan

**Affiliations:** ^1^Department of Clinical Medicine, Faculty of Medicine, Cairo University, Cairo, Egypt; ^2^Department of Optometry, Western University College of Optometry, Pomona, CA, United States; ^3^Department of Clinical Medicine, School of Medicine, University of Jordan, Amman, Jordan; ^4^Department of Ophthalmology, Islamic Hospital, Amman, Jordan; ^5^Department of Nursing Education and Research, Hamad Medical Corporation, Doha, Qatar

**Keywords:** ophthalmology, Monkeypox, Ebola, Zika, MERS, H1N1, influenza, COVID-19

## Abstract

Viral pandemics often take the world by storm, urging the medical community to prioritize the most evident systemic manifestations, often causing ocular manifestations to go unnoticed. This literature review highlights the ocular complications of the Monkeypox, SARS-CoV-2, MERS, Ebola, H1N1, and Zika viruses as the most recent viral pandemics. Research into the effects of these pandemics began immediately. Moreover, it also discusses the ocular complications of the vaccines and treatments that were used in the scope of the viral pandemics. Additionally, this review discusses the role of the eye as an important route of viral transmission, and thereafter, the International recommendations to reduce the incidence of viral transmission were mentioned. Lastly, this paper wants to lay out a platform for researchers who want to learn more about how viruses show up in the eye.

## Introduction

Many viral pandemics have taken their toll on the world throughout the history of humankind. According to the World Health Organization (WHO), more than 23 viral outbreaks are either considered active or are prone to becoming pandemics (1). Thirteen of these pandemics are considered active in the eastern Mediterranean region (2). These pandemics affect various physiological systems in the body, which may spread to many other systems related to the invasion site of the illness. Ocular manifestations of any viral outbreak are an essential aspect of its pathology. Flu, Ebola, and Cholera are all illnesses that occasionally present with ocular symptoms, even though they are not directly related to the eye (3–5).

Immune-mediated or systemic diseases' complications appear to have a close relationship with the eye. The eye, being one of the most sensitive organs in the human body, is often prone to direct infection or to immune-mediated complications, especially in its immune-privileged delicate parts like the Uvea. A few researchers investigate this issue (6).

**Figure 1:**
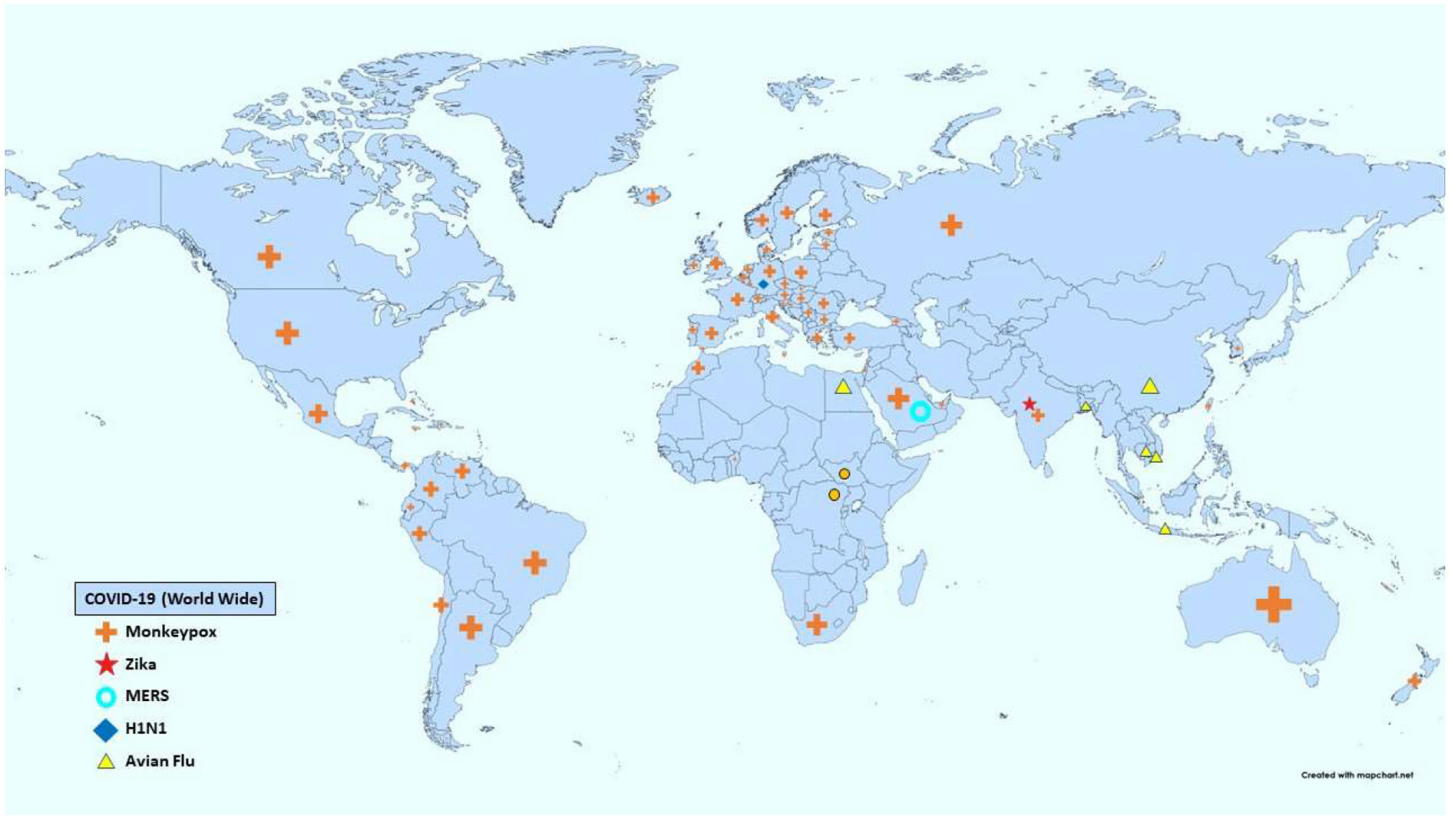
Distribution of viral activity of each pandemic worldwide (12).

The American Centers for Disease Control and Prevention (CDC) and the WHO have several lists categorizing viral pandemics according to various criteria (7, 8). The most recent significant additions to both lists are the Ebola, novel COVID-19, and newly emerging Monkeypox viruses. The WHO list of emergencies included MERS, Zika, and Influenza A subtypes H1N1. Table 1 summarizes the diseases corresponding to each viral spread, their characteristics, and their main systemic manifestations, while Figure 1 provides a map showing the activity of each pandemic according to the CDC as of the July 19th, 2022.

**Table 1:** WHO's emergency viral pandemics.

**Disease**	**Viral family**	**Largest outbreak**	**Country of emergence**	**Zoonotic origin**	**Main systemic manifestation**
COVID-19	Coronaviridae	2019-now (Global)	China	Bats	Upper respiratory tract infection (URTI), Deep vein thrombosis (DVT), acute coronary syndromes (ACS), Guillain-Barré syndrome
MERS	Coronaviridae	2012 (Saudi Arabia)	Middle east (Saudi Arabia)	Bats	Severe respiratory distress syndrome, fatal pneumonia, lower respiratory tract infection
Monkeypox	Orthopoxvirus	2022 (Global)	Travelers from nigeria *non-traveler cases as well (9)	Unknown (10)	Fever, intense headache, lymphadenopathy, back pain, myalgia, intense asthenia, skin rash
Flu: H1N1	Orthomyxo-viridae	2009–2010 (Global)	Mexico	Pigs	Pneumonia, ARDS, GIT disturbance: Vomiting and diarrhea
Zika	Flaviviridae	2015–2016 (Global)	Uganda	Rhesus Monkeys	Guillain-Barré syndrome (GBS), Congenital Zika syndrome: Microcephaly and many malformations.
Ebola	Filoviridae	2014–2016 (West Africa DRC) (11)	Democratic Republic of the Congo (DRC)	Fruit bats of the Pteropod-idae family	Fever, fatigue, muscle pain, headache, sore throat, vomiting, diarrhea and rash, impaired kidney and liver function, internal and external bleeding, hemorrhagic fever

In this review of the literature, our team highlights the most common ocular symptoms of the most recent pandemics. In addition, this review is intended to provide an easy access to ocular manifestations induced by the world's recent pandemics as well as a beneficial resource to those interested in ocular health and systemic virulence of such infectious diseases for future research directives and further comprehensive reviews.

This topic was chosen as a trial to highlight the ocular complications of recent active viral pandemics. As most viral pandemics affect the respiratory system and cause death from lung affection, ocular manifestations and complications of these pandemics often go under-reported by reviews, and thus, light needs to be shed on this topic, especially because ocular complications may result in permanent morbidity and low quality of life for patients. A comprehensive online literature search was conducted using the keywords “ocular complications,” “ocular manifestations,” and “eye complications” as primary terms. Then we added each keyword to the following keywords: “ZIKA,” “MERS,” “monkeypox,” “SARS-CoV-2,” “Ebola,” “influenza A,” and “influenza h7n9,” on PubMed, Embase (Elsevier), and Cochrane library. The identified studies were screened by title and abstract to be up-to-date and related to our topic. The data extraction was done in anatomical arrangement, including each anatomical structure separately to ensure coverage of all eye structures.

## Monkeypox

On the 23rd of July 2022, the WHO announced the Monkeypox disease as an emergency after having spread to 72 countries worldwide (13). Monkeypox disease is caused by the monkeypox virus, a double-stranded DNA member of the Orthopoxviral genus in the family Poxviridae. It was first identified in humans in 1970 in the Democratic Republic of the Congo in a 9-month-old boy. However, it has been known as a disease of monkeys since 1958, hence the name. Since the eradication of smallpox in 1980, it has emerged as the most important Orthopox virus for public health (13, 14).

After 1970, several outbreaks of monkeypox were reported, mostly in central Africa, until 2003, when the United States of America announced a monkeypox outbreak linked to contact with pet prairie dogs. Since 2017, Nigeria has experienced a large outbreak, and in May 2022, several cases of monkeypox infection were reported in non-endemic countries worldwide (13). Zoonotic transmission can occur as well as human-to-human transmission (15). Close contact with respiratory secretions, skin lesions of an infected person or recently contaminated objects can cause transmission. Droplet respiratory particles could cause transmission if prolonged face-to-face contact occurred; however, air-borne transmission cannot be fully ruled out (16). Vertical transmission between mother and fetus can occur before, during, and after birth. Mostly, monkeypox is transmitted by close physical contact. Even though Monkeypox is not considered a sexually transmitted disease, it is often transmitted through sexual contact (not yet fully understood) (14, 17).

Smallpox vaccines can protect against monkeypox, and newer vaccines have been developed, one of which has been approved for the prevention of monkeypox. Cessation of smallpox vaccination is suspected to render communities to become more vulnerable to monkeypox. Usually, monkeypox is self-limited, with symptoms clearing in 2–4 weeks. Severe cases can occur with mortality rates around 3–6% (13). Monkeypox clinically resembles smallpox, but it is less contagious, and it causes less severe symptoms. Initially, patients present with fever, intense headache, lymphadenopathy, which are distinctive to monkeypox, along with back pain, myalgia, and intense asthenia. Skin eruptions usually begin within 1–3 days after the onset of fever. The rash affects the face, palms of the hands, soles of the feet, oral mucous membranes, genitalia, and conjunctivae, as well as the cornea. It evolves sequentially from macules to papules, to vesicles to pustules until it turns into crusts that dry up and fall off. Infection is usually more severe among children and is related to the extent of virus exposure, patient health status, and nature of complications. Complications include secondary infections, bronchopneumonia, sepsis, encephalitis, and infection of the cornea with ensuing loss of vision (13, 14).

Ocular complications in monkeypox infection occur in 4–5% of cases (18–20). Eyes are most commonly affected in immunocompromised patients, pregnant women, and children under the age of eight (21). Since monkeypox virus is thought to spread through surfaces by getting into the body through cracks in the skin, the respiratory tract, or mucosal membranes, it is likely that the eye is one way it gets into the body (22). Upon examining rash characteristics in patients infected with monkeypox, 1% of patients had ocular mucosal rash (23).

Eyes' mucosal surfaces are infected early. Conjunctiva, being the outermost mucosal surface of the eye, is vulnerable to monkeypox infection. Cases of acute viral conjunctivitis were reported with monkeypox infections extending to the eyelid to cause blepharitis (23–26). In rat models, ocular viral shedding and ocular discharge were detected, beginning after 5 days of infection, reaching a maximum around 11 days of infection, and gradually ceasing about 21 days after infection. This raises suspicion as to whether the virus can impose a greater risk to the eyes (27).

There is a debate in literature about the origin of these pathologies, as to whether the virus itself causes corneal disease or secondary superimposed bacterial infections are the cause. In many cases, corneal infections were recorded as a complication of monkeypox disease (22). Corneal infection could be exacerbated to corneal pitted scarring and ulceration, which are often associated with serious eye damage and opacity, and even permanent loss of vision (22, 28–31). In cases of secondary bacterial superinfections, corneal perforation, anterior staphyloma, and phthisis bulbi were recorded in correlation with monkeypox (29). An older case of monkeypox that caused serious eye problems had to be treated with a corneal transplant (28).

Early detection and management of systemic monkeypox are important in order to protect the eye from permanent damage. Prophylactic eye protection is recommended when dealing with infected people, especially for medical staff and close contacts (32). However, if infection with monkeypox is confirmed, lubricants should be applied to the eyes in order to prevent complications and protect against blindness. Also, patients should take vitamin supplements to improve their immunity and facilitate viral clearance using natural immune defenses (29). Preservative measures should be taken by physicians to protect patients' eyes in cases of monkeypox, especially since there is a risk of irreversible scarring of the cornea that could lead to permanent loss of the cornea and eventually vision.

Treatments for ocular manifestations in monkeypox patients are still controversial. It is suggested that the use of topical steroids, which reduce inflammation, could precipitate secondary infections and corneal perforations (29). Other medications that were suggested for the treatment of monkeypox were linked to ocular disease as well. IV Cidofovir, an antiviral agent that selectively inhibits viral DNA synthesis, is not to be used as an intraocular injection. Vaccinia is a member of the Orthopox family of viruses and it was used to synthesize the smallpox vaccine (33). Several complications could emerge from the use of the vaccinia vaccine, especially as it is being suggested for monkeypox. Vaccinia Immune Globulin (VIG) is a drug commonly used to treat these complications, but it is contraindicated to use VIG against vaccinia vaccination against isolated keratitis (32).

## Coronavirus disease 2019

Coronavirus disease (COVID-19) is caused by SARS-CoV-2. It emerged in Wuhan, China in late December 2019 (34). It then rapidly disseminated all over the world, with a global estimation of 586 million confirmed cases and 6.425 million deaths have been reported by the World Health Organization at the time of writing this paper (34, 35). SARS-CoV-2 belongs to the Coronaviridae family, which has a single-stranded, positive-sense RNA genome (36). Two modes of transmission of COVID-19 are present: direct and indirect. The direct mode includes transmission by aerosols or respiratory droplets, body fluids as in saliva, urine, semen, tears, and transmission from mother-to-child. Indirect transmission can occur through surfaces in an infected patient's immediate environment as well as objects used on or by the infected person (37).

The majority of SARS-CoV-2 infections present with mild to moderate symptoms. The respiratory system is the most commonly affected system in people infected with SARS-CoV-2. However, increasing data suggests multi-organ systems may be affected. The virus binds to angiotensin converting enzyme 2 (ACE2) receptors in the lungs, heart, brain, vascular endothelial cells, kidneys, intestine, eye surfaces, and other tissues (38).

Patients might present with mild upper respiratory tract infections, cough, sore throat, pneumonia, extending to acute respiratory distress syndrome in severe cases. Pulmonary edema and diffuse alveolar injury with the formation of hyaline membranes have also been observed (39). Cardiac manifestations including myocardial injury, acute coronary syndromes (ACS), cardiomyopathy, and arrhythmias; including tachycardia, bradycardia, and asystole, have been observed with COVID19 infection. Myocardial injury occurred in 20–30% of hospitalized patients with COVID-19, with higher rates (55%) among those with pre-existing cardiovascular disease (40). Coagulopathies are due to a direct effect of SARS-CoV-2 on the ACE2 receptors present on the endothelial cells, inducing endothelial dysfunction, venous thromboembolic disease (VE), deep vein thrombosis (DVT), and pulmonary embolism (PE). The D-dimer level increases in COVID-19 patients, indicating poor prognosis. The source of the elevated D dimer is fibrinolysis and endothelial cell damage due to inflammation (41). These coagulopathies frequently manifest as late manifestations of infection (42).

Hypertension and diabetes were the most common comorbidities associated with severe complications of COVID-19 infection. That was reported in a review on COVID-19-related cardiovascular diseases. It showed that most of the hospitalized patients with COVID-19 had hypertension and diabetes as main comorbidities (40). Diabetes is a risk factor that predisposes to a severe form of many viral diseases, such as influenza A, SARS, and MERS. This could be caused by a number of biological problems, such as high levels of inflammatory biomarkers, high levels of tissue enzymes, and problems with blood clotting (like high levels of D-dimer) (43).

Mild neurological symptoms are also reported in hospitalized patients with COVID-19, including headache, dizziness, myalgia, fatigue, anorexia, anosmia, and ageusia (44). Meningo-encephalitis and acute necrotizing encephalopathy, including the brainstem and basal ganglia, have been described in case reports (45). Renal affection in the form of acute kidney injury (AKI) is the most significant renal manifestation. Hematuria and proteinuria were observed as complications of COVID-19. Patients with chronic diseases, such as diabetes mellitus (DM), may have a higher risk of kidney function deterioration with COVID-19 along with a high mortality rate (40). Ocular symptoms of SARS-CoV-2 infection include redness, tearing, a feeling like there is something in the eye, photophobia, itching, blurred vision, burning sensations, lid margin hyperemia, crusted eyelashes, Meibomian orifice abnormality, follicular conjunctivitis, chemosis, and episcleritis (46, 47).

### Anterior segment

The most common ocular manifestation noted in COVID-19 patients is conjunctivitis, and it may be the only symptom in COVID-19 patients. The conjunctival manifestations are follicular conjunctivitis, conjunctival chemosis, and conjunctival congestion (46, 48). Ocular symptoms such as hyperemia, discharge, and dry eyes are also reported. The virus was detected in tears and conjunctival samples, implicating the eye as a potential route for viral entry (49). The positive rate of SARS-CoV-2 detection in patients' conjunctiva is around 3.9% (50). The American Academy of Ophthalmology (AAO) has recommended replacing the contact lenses with glasses to decrease the risk of viral transmission (51). Keratoconjunctivitis may occur due to direct invasion and inflammation mediated by the virus itself or due to the immune response against the virus. A Chinese case report observed a COVID-19 patient who presented with redness and discharge limited to the left eye. The conjunctival swab tested positive for COVID-19 by polymerase chain reaction (PCR), which was resolved with topical levofloxacin and sodium hyaluronate. The infection resolved and the swab test was negative. However, the patient presented with keratitis and bilateral corneal staining five days later, even though the swab was negative for COVID-19, HSV, and adenovirus. Interlukin-6 was 10 times higher in the left eye. This relapse with bilateral spread and a high cytokine level suggested that the virus induced an immune-mediated keratoconjunctivitis. The patient responded well to fluorometholone. A follow-up with topical corticosteroids is recommended in those patients. Hemorrhagic and pseudomembranous conjunctivitis are also reported (46, 50). Episcleritis is also reported in some cases of SARS-Co-V-2 infection. Ocular manifestations in the eyelids are in the form of Meibomian gland orifice abnormalities accompanied by lid margin hyperemia or telangiectasia. Blepharitis is associated with COVID-19 disease onset and offset since it develops as a delayed consequence of the infection. The number of cases is also expected to rise after the pandemic, especially in people who already had changes to the surface of their eyes (52).

A study from two major hospitals in Egypt examined 228 COVID-19 patients for ocular symptoms. Conjunctivitis was found in 34 of them (14.9%), manifesting with redness, foreign body sensation, and epiphora (53). In Qatar, upon studying ocular manifestations of COVID-19 in 500 patients, 39 (7.8%) presented with eye manifestations varying between hyperemia, eye pain, epiphora, burning sensation, and photophobia (47).

### Posterior segment

Central retinal vein occlusion (CRVO) and central retinal artery occlusion (CRAO) are two of the many vascular manifestations of COVID-19. Patients with COVID-19 have hyper-coagulability as evidenced by higher levels of fibrinogen, prothrombin time (PT), prothrombin dimer, and activated partial thromboplastin time (aPTT). SARS-CoV-2 infection has also been associated with paracentral acute middle maculopathy (PAMM) and acute macular neurocristopathy (AMN). However, the relationship between these conditions and COVID-19 remains unknown. Optical coherence tomography (OCT) has shown hyper-reflective lesions at the level of ganglion cells and inner plexiform layers prominent at the papilla-macular bundle in both eyes in some patients (46). The commonest retinal features are cotton wool spots, micro-hemorrhages, hard exudates, and tortuous veins. The mechanism through which SARS-CoV-2 may affect the retinal vasculature is poorly understood. It is known that ACE-2 receptors are abundant in the retina and choroid (54). Choroid thickness is thought to be reduced in the early post-infectious period of the COVID-19 disease. The retinal pigment epithelium, which is in close proximity to the choroid and shares certain antigenic epitopes with SARS-CoV-2, may cause localized inflammatory damage to the choroid in susceptible individuals. Choroidopathy is likely caused by this systemic inflammation. Studies have shown that choroidopathy changes are reversible as initial thickness of the choroid increased 9 months after infection by COVID-19 (55).

### Kawasaki disease in children and COVID-19 infection

Kawasaki disease is a type of vasculitis that is self-limiting, and is commonly accompanied by conjunctival injection, punctate keratitis, vitreous opacities, papilledema, and subconjunctival hemorrhage (56). An Italian study provided evidence of strong correlation between COVID-19 infections and an outbreak of Kawasaki-like disease (KD). Eighty percentage of children with positive COVID-19 serology showed a 30-fold increase in incidence of a severe form of KD (57, 58). The goal of treatment is to reduce systemic inflammation using corticosteroids, intravenous immunoglobulin (IVIG), and aspirin as per case reports (59).

### Ocular adverse effects after COVID-19 drug administration

Chloroquine (CQ) and hydroxychloroquine (HCQ) are used for the treatment of malaria and some autoimmune diseases such as rheumatoid arthritis and systemic lupus erythematosus. Recently, studies have proven the effective action of CQ and HCQ against the SARS-CoV-2 virus. CQ and HCQ were widely used during COVID-19 pandemic with positive results in preventing pneumonia, improving the pulmonary imaging results and promoting virus-negative seroconversion (60). These drugs have ocular complications and other systemic adverse effects. On screening for CQ and HCQ retinopathy, the American Academy of Ophthalmology recommendations suggest keeping a daily dosage below 2.3 mg/kg in patients receiving CQ and <5.0 mg/kg in those using HCQ. However, most patients who receive CQ and HCQ for COVID-19 treatment receive a considerate amount for a long time which is toxic to the retina (56).

HCQ and CQ ocular toxicity may present with a bilateral maculopathy characterized by a ring of parafoveal RPE depigmentation that initially spares the fovea among other symptoms like posterior subcapsular lens opacity, ciliary body dysfunction, and whorl-like corneal intraepithelial deposits, that are usually reversible (61).

In advanced cases of maculopathy of CQ and HCQ origin, progressive loss of visual acuity, RPE atrophy with foveal involvement, and a widespread photoreceptor loss manifest. A maculopathy of HCQ and CQ origin is usually irreversible, and it may progress regardless of drug cessation. This could be attributed to the metabolic injury inflicted on retinal cells during drug exposure, causing a gradual decompensation of these cells (61).

### Lopinavir*/*ritonavir combination

These are antiretroviral medications of the second generation, used to treat HIV patients. In the literature, there was a potential effect on COVID-19 viral load reduction using this combination of drugs. Generally, the clinical picture consists of pigmentation changes in the macula. These changes may present as a bull's eye or a granular pattern, or with less specific patterns, and could even lead to severe vision loss (56).

Crystalline intraretinal deposits and pigment alterations that resemble bone-spicules in the mid-peripheral retina can also occur. Macular thinning with atrophy of the outer retinal layers, loss of the ellipsoid zone, and aberrant hyperreflectivity are OCT findings in such cases. Only persistent usage of ritonavir has been linked to retinal toxicity (56).

Research reports an average waiting period of 19 months before diagnosis. Patients with COVID-19 are advised to take 400/100 mg of lopinavir/ritonavir twice day. The typical course of therapy for COVID-19 is 5–7 days (62). Therefore, it is unlikely that COVID-19 patients will experience retinal damage from short-term lopinavir/ritonavir therapy. IFN-beta-1, interleukin-1 inhibitors (such as Anakinra), and interleukin-6 inhibitors are examples of immunomodulatory medications (e.g., sarilumab, siltuximab, and tocilizumab). The use of these medications in COVID-19 patients may be advantageous, according to some studies (56). In the first phases of the infection, IFN-beta-1 can also be employed (63). IFN-beta-1 can cause cotton-wool patches, retinal hemorrhages, and other anomalies in the retinal microvascular system in the eyes, which are, however, reversible.

Cytokine storm is attenuated by interleukin-1 and interleukin-6 inhibitors (61). Nystagmus is associated with high doses of anakinra, and bilateral papilledema, HTLV-1 uveitis, viral conjunctivitis, and ophthalmic herpes zoster infection are associated with tocilizumab and tocilizumab-associated bilateral retinopathy with multifocal cotton-wool spots and retinal hemorrhages, respectively (56).

### Post COVID-19 vaccination

Many vaccines are developed against the COVID-19 virus and a handful are approved for use which in turn forms immunity against the virus. However, these vaccines have ocular and systemic side effects. A narrative literature review about the ocular adverse events after COVID-19 vaccination reported the following side effects: facial nerve palsy, abducens nerve palsy, acute macular neuro-retinopathy, central serous retinopathy, thrombosis, uveitis, multiple evanescent white dot syndrome, Vogt-Koyanagi-Harada disease reactivation, and new-onset Graves' disease. Literature hypothesizes that the immune response to the COVID-19 vaccination can be the cause of the ocular side effects after COVID-19 vaccination (64). A systematic review conducted by (Yu-Kuei Lee and Yi-Hsun Huang) regarding the ocular manifestations post-COVID-19 vaccinations reported the following side effects: Eyelid manifestations including eyelid swelling, purpuric lesions and Herpes Zoster Ophthalmicus (HZO). Corneal affection manifested by graft rejection after corneal surgery like penetrating keratoplasty (PKP) and Descement's membrane endothelial keratoplasty (DMEK). Acute anterior uveitis, panuveitis, multifocal choroiditis, acute zonal occult outer retinopathy (AZOOR) and reactivation of Vogt- Koyanagi-Harada (VKH) disease were reported. The retina was affected by central serous retinopathy, acute macular neuro -retinopathy (AMN), and retinal detachment. Optic neuritis, arteritic anterior ischemic optic neuropathy (AAION) and abducens nerve palsy were also reported. Vascular complications manifested by including superior ophthalmic vein thrombosis, cerebral venous sinus thrombosis, thrombocytopenia, acute ischemic stroke and bleeding (65). Although the present data reports many systemic and ocular side effects, people are encouraged to take the vaccination as its benefits outweigh the risks.

## Middle East respiratory syndrome coronavirus

MERS is another member of the Coronaviruses family. Middle East respiratory syndrome coronavirus (MERS-CoV) is the pathogen responsible for the outbreak of the severe respiratory disease in the Middle East in June 2012, resulting in 2,494 infections of whom 858 died, with 20–40% mortality rate (66, 67). The first case reported was from Jeddah, Saudi Arabia, then the infection spread to countries around the Arabian Peninsula. According to the WHO, 27 countries have reported cases of MERS (68, 69). MERS-CoV is a zoonotic virus, believed to have originated in bats and transmitted from bats to humans through Dromedary camels. The clinical picture of MERS infection ranges from asymptomatic infections to flu-like symptoms, up to severe respiratory distress, and fatal pneumonia. Acute renal impairment was the most striking feature of MERS infection, which is a unique symptom of MERS infections since it was not seen in other COV infections. Other symptoms consist mainly of lower respiratory tract infections, including fever, cough, chills, sore throat, myalgia, and arthralgia (69).

Conjunctivitis was the only ocular manifestation noted in MERS-CoV. It was reported in a study conducted in Makkah that only 6 patients out of 261 (2% of cases) suffered from conjunctivitis (70, 71). Although no ocular complications other than conjunctivitis were reported in the case of MERS virus infection, other studies showed the potential for detecting the viral genome in tears and conjunctival secretions, which could aid viral transmission (67, 72).

## Influenza A viruses

Influenza A is a group of viruses classified into 16 hemagglutinin (HA) subtypes and 9 neuraminidase (NA) subtypes (73). In addition, Influenza A viruses are further divided into low pathogenic avian influenza (LPAI) and high-pathogenic avian influenza (HPAI) viruses depending on their pathogenic properties in chickens (74).

In early June 2009, the WHO declared the H1N1 virus a pandemic according to the criterion that the transmission was intercontinental (75). The virus originated as a consequence of the incorporation of genes between swine, avian, and human viruses, leading to the term “swine flu” which affects humans. The infection was in the form of a typical lung infection that caused mild disease but occasionally led to acute respiratory distress syndrome (ARDS) and death (76). As a result of the rapid spread all over the world, nearly 214 countries were affected. The total reported cases were close to 700 million to 1.4 billion cases. Over 18,000 deaths were estimated to have been reported to WHO. This flu virus caused severe morbidity but only had a 1–4 percent death rate (76).

The Centers for Disease Control and Prevention (CDC) defines cases as influenza-like illness (ILI) if there is a mild illness like cough, sore throat, diarrhea, myalgias, or headache. Vomiting and diarrhea have been reported in some patients, but no shortness of breath, dyspnea, or severe dehydration. Pneumonia was the most common and serious complication of the 2009 H1N1 pandemic influenza, while throat congestion and swollen tonsils were the most common systemic signs (77). One of the most common complications of the 2009 swine-flu is the secondary bacterial infection with Streptococcus pneumonia, which was the most common bacteria identified. The fetal outcome, morbidity, and ICU needs appear to have occurred due to secondary bacterial infections as well (78).

H1N1 could replicate in the human conjunctiva, making the ocular surface an important route of infection. In addition, the virus was also isolated from other ocular tissues like cornea, trabecular meshwork, and RPE cells (79). It nearly affects all eye parts starting with the conjunctiva causing direct conjunctival invasion by the influenza virus and presents most commonly as bilateral acute conjunctivitis in 58 cases (65%). Acute conjunctivitis was linked to significant eyelid edema, conjunctival hyperemia, watery discharge, and moderate chemosis. As to the cornea, 18 cases presented with bilateral multiple corneal erosions that subsided by the 7th day of the illness (79).

Regarding the posterior segment of the eye, retinopathy, retinitis, angiitis, uveal effusion syndrome, acute posterior multifocal placoid pigment epitheliopathy (APMPPE), and serous macular detachment (SMD). SMD is observed in cardiovascular disorders, and excessive vascular permeability of retinal and/or choroidal circulation and retinal pigment epithelial (RPE) damage as the serous macular detachment happens most likely due to blood-retinal barrier damage by the circulating immune complexes or direct virus replication in the patient (78). Inflammation of the choriocapillaris with subsequent atrophy of the retinal pigment epithelium (RPE) has also been reported in rare cases, causing destruction of the blood-retina barrier that will disrupt the immune privilege of the eye (80).

Other observations were lesions in 3 patients presented with disc and intra-retinal hemorrhages. The disc hemorrhages and macular ischemia are consistent with arterial obstruction and retinal ischemia along with systemic thrombotic events in the form of cerebral hemorrhage and thrombus in radial artery. However, the authors reported that the thrombocytopenia or bleeding predisposition did not seem to be the cause of these bleeds, suggesting other mechanisms (81).

Ocular complications manifesting after influenza vaccination include mild and moderate conjunctivitis and a slight decrease in visual acuity up to a visual loss in two cases after receiving the vaccination. Also, the anterior segment was normal, but the fundus examination revealed bilateral optic swelling with venous engorgement. Some literature attributes the visual loss to immune complex-mediated vasculopathy by causing anterior ischemic optic neuropathy (82). Another study reported the diagnosis of an altitudinal visual field defect associated with segmental disc swelling and visual loss, which was bilateral and associated with systemic symptoms and raised serum inflammatory markers. These observations were made in a 68-year-old male patient 10 days after receiving the vaccine. Over the next 3 months, his visual acuity in the left eye recovered to 6/36 with a persistent field defect (83).

### H7N9 pandemic and H5N1

Human infections with H7 influenza virus subtypes were reported in Italy, the Netherlands, Canada, Mexico, the United States of America, and the United Kingdom (84). The first time that a human infection with the influenza A (H7N9) virus has been identified in China, after three people who were seriously ill were confirmed to have been infected. Three outbreak waves in humans then followed (85). The first outbreak emerged in February 2013, and then the virus ceased rapidly after April 2013. The second wave began in October 2013 and also ceased in February 2014. The number of H7N9 cases increased again in late 2014 and peaked in January 2015 during the third wave (86). Between March 25 and September 31, 2013, a total of 134 cases of H7N9 influenza infection were identified, and by the end of September 2015, a total of 17 provinces or municipalities had been affected in Mainland China and a total of 656 H7N9 cases had been reported, 268 of which were fatal.

General symptoms of the illness included fever and cough (which were the most common symptoms), shivering, fatigue, muscular aches, nausea, and vomiting (84). However, there was a notable absence of conjunctivitis cases during the first wave of human infections with H7N9 viruses in China. This indicates that the presence of an H7 hemagglutinin might be required for ocular tropism, but this is not sufficient to confer this property. For the time being, the factors that enable certain H7 viruses to infect and replicate in the eye remain unclear (85). One study compared the capability of influenza viruses to replicate in the epithelium of the human cornea. It found that H7N9, HPAI, H5N1, and seasonal H3N2 viruses showed no ocular symptoms and are considered respiratory or non-ocular-tropic (85).

## Zika

Zika virus was discovered in 1947 in Uganda. It caused an outbreak in 2015 in Brazil, affecting more than 89 countries worldwide by early December 2021 (87, 88). It is characterized by having an envelope and a single-stranded RNA positive sense, and it belongs to the Flaviviridae family of viruses. It is considered an arthropod-borne virus, which means that the transmission occurs by arthropod vectors, specifically the Aedes mosquito's genus. However, there are many other routes of transmission from human to human, such as sexual contact, blood transfusions, and from mother to fetus. Also, ocular transmission was reported to occur when there is contact with conjunctival fluid and tears. Studies have suggested that contact with ocular discharge, such as tears of a patient with Zika virus infection, can act as a non-vector mode of transmitting Zika virus. The persistence of Zika virus RNA in tears 30 days post-infection has been documented. Thus, there is a potential risk of ocular transmission of Zika virus through contact with Zika virus-infected tears (89).

The primary manifestations range from an asymptomatic and mild flu-like illness to severe manifestations such as microcephaly in babies and Guillain-Barre syndrome in adults (90). However, it is important to recognize the differences between the manifestations of congenital and non-congenital forms of Zika virus infection. Asymptomatic cases make up more than 75% of the total cases. While the symptomatic cases presented by symptoms include maculopapular rash, arthralgia, and conjunctivitis. Retro-orbital pain and headache are also observed. In newborns, it causes microcephaly, which is a neurologic condition characterized by a small head size due to impaired development of the brain of a baby. A relationship between Zika virus and microcephaly was noticed in 2015 in Brazil as Zika virus was detected in the amniotic fluid of fetuses who were born with microcephaly. Evidence-based studies have supported this association. There is a case-control study that showed that 12 out of 32 newborns with microcephaly were infected with zika virus (91). In adults, the main important clinical symptom was Guillain-Barré syndrome (GBS), which causes damage to the nerve cells, leading to muscle weakness that may even lead to paralysis (92).

On the anterior segment in newborns, Zika causes iris coloboma, lens subluxation, cataract, glaucoma, microphthalmia, and intraocular calcifications. Anatomical abnormalities such as pupillary membrane and mal-development of the anterior chamber angles are all reported in Congenital Zika Syndrome (CZS) (89). In adults, non-purulent conjunctivitis with hyperemia is the most common manifestation of acute Zika virus infection as it arises in about 63% of patients. Keratitis, and elevated intraocular pressure (IOP), which is a risk factor for glaucoma, were also reported (93).

In the posterior segment in newborns, Zika virus infection has been shown to damage the posterior segment of the eye, including the retina, optic nerve, and retinal vessels. Pigment mottling and chorioretinal atrophy are the most visible ocular findings in CZS. It often mimics toxoplasmosis, macular abnormalities, chorioretinal scarring, retinal hemorrhage, vascular tortuosity, and washed-out peripheral retina. Zika virus's ability to cross the retinal barrier allows it to infect the retinal cells. It manifests as chorioretinal atrophy and changes in the pigmentation of the retinal pigment epithelial cells, which appear as mottling of the retinal pigment epithelium. The inflammation causes damage to the blood-retinal barrier cells and the adjacent choroid (94). In adults, reported manifestations were similar to those of newborns involving chorioretinitis, multifocal choroiditis, chorioretinal atrophy, mottling of the retinal pigment epithelium, and macular pigment mottling (93).

Abducens nerve and oculomotor paresis were reported as neuro-ophthalmic complications which were presented by convergent strabismus and squint. Optic disc hypoplasia with the double-ring sign, disc pallor, and enlarged cup-to-disc ratio were also reported. In adults, papilledema, ophthalmoplegia, and ocular flutter were the main neuro-ophthalmic complications (89).

## Ebola

The Ebola virus is a ssRNA member of the Filoviridae viral family, falling under the genus of Ebolavirus. Ebola virus disease (EVD), or Ebola hemorrhagic fever, is an acute viral infection that is often fatal, having a mortality rate average of 50%, ranging from 25 to 90% in past outbreaks. It affects humans and other primates and is transmitted to humans from wild animals or through human-to-human transmission. EVD appeared for the first time in 1976 in the Democratic Republic of the Congo (DRC) near the Ebola River, from which the disease got its name. In the same year, another outbreak occurred in South Sudan. The largest EVD outbreak took place in West Africa during the period of 2014–2016 (95).

Fruit bats in the Pteropodidae family are thought to be the natural hosts for the virus. Humans can acquire the organism through the blood, secretions, and bodily fluids of infected animals like fruit bats, gorillas, chimpanzees, etc., which can be dead or ill in the rainforest. Human-to-human contact then occurs through direct contact with blood or other bodily fluids of infected patients or with contaminated objects with bodily fluid (blood, vomit, feces) of sick or dead individuals. The incubation period of EVD varies between 2 and 21 days, and the risk of transmission starts with the first appearance of symptoms. Fever, fatigue, muscle pain, headache, and sore throat are the first symptoms to appear. Consequently, vomiting, diarrhea, and a rash follow. Symptoms of impaired kidney and liver function may appear, as well as symptoms of internal and external bleeding. Diagnosis of EVD can be confused with similar diseases like typhoid fever, but it can be of critical importance in cases of pregnancy. Therefore; serology testing like antibody-capture enzyme-linked immunosorbent assay (ELISA) and reverse transcriptase polymerase chain reaction (RT-PCR) is used to confirm an EVD diagnosis (95).

Supportive treatment, mainly rehydration, and symptomatic treatment are the main course of action in EVD patients (Inmazeb and Ebanga) are two monoclonal antibodies that were approved by the US Food and Drug Administration (FDA) in late 2020 for the treatment of Zaire ebolavirus infection in adults and children (96). The Ervebo vaccine was developed to protect against the Zaire ebolavirus and it was approved for the use in adults 18 years or older (except for pregnant and breastfeeding women) by the FDA in 2020 (95, 96).

Ocular symptoms occur during acute EVD until up to 17 weeks after discharge. conjunctivitis, subconjunctival hemorrhages, and acute vision loss of unknown etiology have been reported, especially in the setting of hemorrhagic EVD (97). Conjunctivitis is one of the earliest signs of EBOV infection and has an important prognostic value since it indicates severe disease when hemorrhagic (71, 98). Occasionally, bilateral conjunctivitis can be the primary symptom of EVD; thus, the patient may be presented to an ophthalmologist first.

In the Kikwit (the southwestern part of the Democratic Republic of Congo) epidemic, conjunctivitis was observed in 48% of the patients and was considered a suggestive symptom of acute EBOV infection (97). Blurred vision and blindness with unknown etiology was a complaint of 38% of patients (99). Experts warn that untreated ocular symptoms can lead to vision impairment and blindness (100). Optic neuropathy, ocular motility disorders, episcleritis, interstitial keratitis, cataract, and glaucoma can manifest as complications of acute EVD (101, 102).

Although EVD can cause ocular manifestations in its acute phase. The EVD convalescence phase has been linked to the most serious complications. A post-Ebola virus disease syndrome was identified in EVD survivors. Among several systemic complications ranging from arthralgia and musculoskeletal pain to headaches and psychological problems (103, 104). Uveitis was the most common ocular finding in survivors of EDV (13–34%) (105). The cause of these pathologies is not clear yet, but explain it in terms of immune dysregulation or autoimmunity, especially since the manifestations are highly prevalent in immune-privileged sites (103).

Patients of post-EVD syndrome commonly present with eye pain, photophobia and redness which may lead to acute or chronic vision loss (97, 101). Uveitis could be diagnosed in EVD as early as within 2 weeks of infection. But the highest risk can be suspected within 2–3 months after confirming the diagnosis. Cases were reported of manifested uveitis even after 13 months of a negative RT-PCR (106).

An example of late ocular manifestations of Ebola virus can be given by Mattia et al. (107). This cross-sectional study reported a 46% prevalence of anterior uveitis, 3% intermediate, 26% posterior, and 25% panuveitis among their 227 post-EDV patients. These cases were observed at an average time of 121 days after discharge from the clinic and receiving treatment for acute disease. An important finding is that, Ebola virus was detected in the aqueous humor in some cases of patients with negative PCR tests for the virus in their serum (108, 109).

A large study by the name Ebola Virus Persistence in Ocular Tissues and Fluids (EVICT) was conducted by Shantha et al. in Sierra Leone targeting 50 survivors of EVD, 46 of whom had visually significant cataracts due to EVD, 2 with active uveitis, 1 subluxated lens, and 1 blind painful eye due to uveitis. Upon testing their aqueous humor for EBOV RNA using RT-PCR at a median of 19 months, none was found in any of the patients (105, 110).

A study on the same database of EVICT was performed to assess posterior segment manifestations of EVD was performed on 250 eyes of 125 survivors (111). Other than cataract, posterior synechiae and different types of uveitis, chorioretinal scarring (10%), optic neuropathy (3%), vitreous opacities (3%), retinal detachment (2%), epiretinal membrane (1%), vitreomacular traction (1%) and retinal pigment epithelium atrophy of one eye were detected.

The management of EVD eyes, especially with regards to uveitis, was successfully performed using corticosteroids. Topical corticosteroids and even oral corticosteroids in severe cases were found effective in treating EVD uveitis (100).

## Discussion

This literature review aims to highlight the ocular complications of the most recent viral pandemics. Attention was applied to this topic previously (71, 102), and researchers took an interest in ocular manifestations of certain viral pandemics. This article tackles the topic in the cases of the most recent and currently active viral pandemics.

Many observations were made in this literature review, the first of which was that viral transmission through tears and ocular secretions can pose a serious threat despite the fact that airborne transmission has higher infectivity. Ebola virus was detected in the aqueous humor of one patient, but evidence of viral transmission through tears or conjunctival secretions is still lacking (112). Zika and MRES viruses can be transmitted through ocular secretions, especially conjunctival secretions (89, 113). Influenza A viruses can replicate in the conjunctiva of infected people, which could be its route of access to the respiratory mucosa (79). In COVID-19, the eye is suspected to be a potential route of transmission (50). Some case reports noted the detection of viral DNA/RNA in cases (COVID and Ebola) even though the serum PCR was negative for viral DNA/RNA. Wearing glasses during the examination helped reduce infection susceptibility in many cases.

Ophthalmic manifestations in viral outbreaks varied from mild symptoms to sight threatening and blinding diseases. The most common ocular manifestation was acute conjunctivitis, which was reported in all viral pandemics studied. For example, in COVID-19, a case report from Jordan reported conjunctivitis as the only presentation (48). Some viral infections might progress to keratoconjunctivitis either by direct infection to the cornea or by autoimmune reactions (46).

Glaucomatous disease was observed in Zika and Ebola viral outbreaks. Whether in the context of congenital Zika syndrome, acute Zika infection, or as a component of acute ocular disease in acute EVD. A pregnant mother infected with Zika virus might give birth to a baby with congenital glaucoma as a part of congenital zika syndrome. This syndrome causes more severe ocular affection than acute Zika in newborn babies. Therefore, screening pregnant females for Zika infection and taking eye protective measures for newborn babies of Zika infected moms can be of crucial importance, especially since eye abnormalities may be the first presenting signs of congenital Zika infection (117).

Regarding the posterior segment of the eye, retinal vascular abnormalities and thromboembolic events were the most serious observations. Except for monkeypox and MERS, which were also linked to retinal problems like retinal pigment epithelium (RPE) abnormalities and retinal detachment, this was true for all viral outbreaks that were studied.

An interesting observation was made when comparing the ocular manifestations of the MERS and SARS-CoV-2 viruses, which are members of the same viral family. On one hand, the MERS virus causes a more severe systemic disease and has a higher mortality rate. However, it only causes mild ocular complications, usually limited to conjunctivitis. COVID-19 disease, on the other hand, is less aggressive and is linked to a milder form of systemic disease and a lower death rate. However, its eye symptoms are more serious and could lead to damage to the optic nerve or even blindness.

One part of the ocular manifestations of viral pandemics that often goes understudied is the ocular complications imposed by their vaccines. In some cases, H1N1 vaccines were linked to conjunctivitis and visual acuity deterioration. In other cases, these effects were linked to immune-mediated vasculopathy. Moreover, the novel COVID-19 vaccines were associated with several symptoms that ranged from eyelid swelling to facial nerve paralysis and optic neuritis. The causes of these effects were unexplained and variable. Hence, the literature hypothesizes that the immune response to the COVID-19 vaccination may be the cause of the ocular side effects observed (65). Although the present data reports many systemic and ocular side effects, people are also encouraged to take the vaccination as its benefits outweigh the risks.

**Figure 2:**
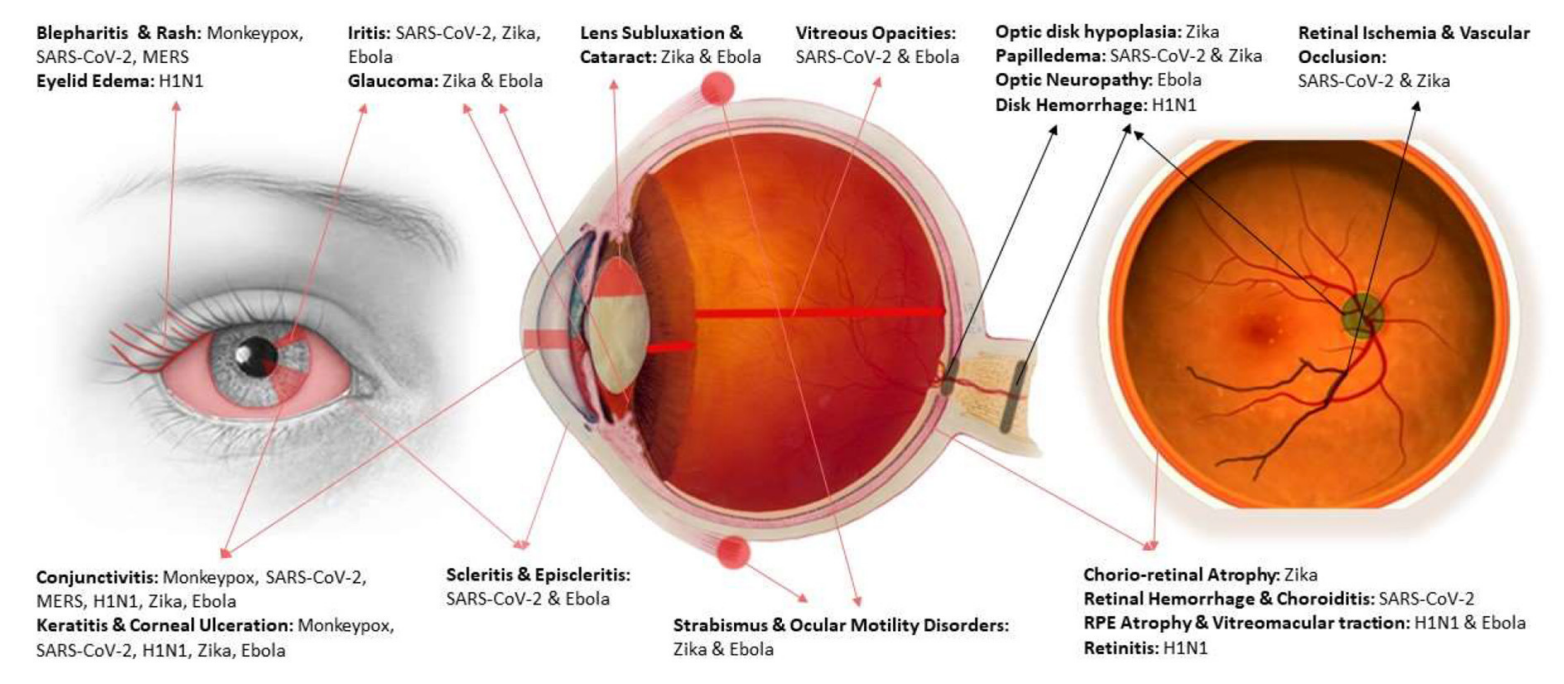
An illustration for affected structures by each viral disease (114–116).

An important message to take from this review would be the necessity of protective and prophylactic measures for optometry and ophthalmology professionals who might get involved in the diagnosis and management of patients in viral outbreaks. Adherence to safety protocols and the proper use of protective equipment are substantial factors to consider. Facemasks, face-shields, eye goggles, gloves, and protective gear should be worn by all participating medical staff. Also, using disinfectants and maintaining the equipment's sterility are of significant importance. In addition to the regular personal hygiene and infection containment protocols, the American Academy of Ophthalmology (AAO) has recommended replacing the contact lenses with glasses to decrease the viral transmission. Also, adapting a suitable operations protocol and laboratory management to limit the exposure to disease could help in protecting staff (51, 71). Figure 2 provides a visual illustration for affected structures of the eye and the causative viral disease for each.

While this review provides the reader and researchers with rich information about the prospective viral diseases herein, this review will be a rich resource for researchers who are interested in further investigating eye-related viral manifestations. Data was gathered based on the dependability and authenticity of resources and published works that adhered to the academic professionalism that each of us holds and promotes.

## Conclusion

We conclude that active viral pandemics have many ocular complications. These effects can easily go unnoticed because most researchers focus on the direct systemic complications and leave the ocular manifestations out. The eye may harbor the virus in many viral diseases and may be a route of transmission in many cases. Even when the virus itself does not cause ocular pathologies directly or indirectly, vaccines and medical treatments may play a role in causing ocular manifestations. Thus, protective and prophylactic measures should be taken by optometrists, ophthalmologists, and by the general population, and the highest hygiene standards should be followed in ophthalmology practice in general. Ocular manifestations of viral pandemics are in need of further investigation and research. However, we strongly believe that this thorough review will provide a great platform for those interested in investigating viral diseases, focusing on eye manifestations in particular.

## Method of literature search

A literature search was performed for articles (English) using Medline and Google Scholar, for any date up to July 2022. The following keywords were used “Ophthalmology”, “Ocular”, “Eye”, “Viral pandemics”, “Ebola”, “Zika”, “MERS”, “H1N1”, “Influenza”, “COVID-19”, “Monkeypox”. Search results were initially reviewed by title and abstract, and articles were selected for more in-depth analysis if deemed relevant. Pertinent articles were examined in-depth for this report.

## Author contributions

MT, MA, WA, SS, and YA-B: research design, data collection, literature search, and manuscript preparation. LA-I, TA, and AN: final review. All authors have read and agreed to the published version of the manuscript.

## Funding

Open Access funding provided by the Qatar National Library.

## Conflict of interest

The authors declare that the research was conducted in the absence of any commercial or financial relationships that could be construed as a potential conflict of interest.

## Publisher's note

All claims expressed in this article are solely those of the authors and do not necessarily represent those of their affiliated organizations, or those of the publisher, the editors and the reviewers. Any product that may be evaluated in this article, or claim that may be made by its manufacturer, is not guaranteed or endorsed by the publisher.
